# Qualitative Evaluation of YouTube Videos on Dental Fear, Anxiety and Phobia

**DOI:** 10.3390/ijerph20010750

**Published:** 2022-12-31

**Authors:** Natalie Sui Miu Wong, Andy Wai Kan Yeung, Colman Patrick McGrath, Yiu Yan Leung

**Affiliations:** 1 Oral and Maxillofacial Surgery, Faculty of Dentistry, The University of Hong Kong, Hong Kong; 2 Applied Oral Sciences & Community Dental Care, Faculty of Dentistry, The University of Hong Kong, Hong Kong

**Keywords:** dental fear, dental anxiety, dental phobia, YouTube, non-pharmacological, pharmacological, media, online patient education material

## Abstract

The aim of this study was to review the health information of dental fear-, dental anxiety-, and dental phobia-related videos on YouTube. The 100 most widely viewed videos for the keywords “dental fear”, “dental anxiety”, and “dental phobia” were chosen for evaluation. Out of the 300 videos, 145 videos met the inclusion criteria and were analyzed. It was found that most of them were produced by the professions, with a dentist delivering the key messages or with patients giving testimonials. Many etiological factors and symptoms were described. Many pharmacological and non-pharmacological interventions were recommended to the audience, such as sedation and distraction, respectively. However, there was a lack of information on the definition or diagnostic criteria of dental fear, dental anxiety, and dental phobia. Videos with high views had a higher ratio of misleading information. Videos with a dentist being the informant had a similar ratio of misleading information compared to other videos. Without adequate information on how to diagnose, it would be very difficult for the audience to determine if the video content was relevant or useful. The dental profession can work together with psychologists or psychiatrists to produce authoritative videos with accurate content.

## 1. Introduction

Dental fear and dental anxiety are two terms frequently used interchangeably, commonly under an umbrella term of dental fear and anxiety [1]. It is a common phenomenon and frequently investigated in academia [2,3]. Recent meta-analyses calculated that its global prevalence rate was as high as 15.3% among adults [4] and 25.8–36.5% among preschoolers and schoolchildren [3]. People with higher levels of dental fear and anxiety were found to have lower self-esteem and lower morale [5]. Moreover, higher levels of dental fear and anxiety were associated with an increased number of decayed teeth [6], more severe dental pain, and hence a lower oral-health related quality-of-life [7,8]. Therefore, it is of paramount importance to gauge and manage the dental fear and anxiety of affected patients, so that they can benefit from more relaxed dental visits and smooth dental treatments. There are many psychometric scales available in the academic literature to measure the level of dental fear and anxiety of patients [9], with Corah’s Dental Anxiety Scale (DAS), Kleinknecht’s Dental Fear Survey (DFS), and Dental Anxiety Inventory Short Version (DAI-S) being some of the notable examples. Moreover, many management techniques for dental fear and anxiety have been proposed, ranging from non-pharmacological (e.g., cognitive behavioral therapy, distraction) to pharmacological [10,11]. Furthermore, a recent meta-analysis showed that significant anxiety reductions using non-pharmacological interventions could be demonstrated through psychometric assessment [12]. Such findings implied that dental fear and anxiety is a manageable condition once the patient is able to find appropriate help.

Meanwhile, dental phobia can be considered a more severe condition, as some researchers have suggested that one way to differentiate between dental anxiety and dental phobia is to consider their impact on normal functioning; that is, if they affects the patient’s occupation or social activities [1]. As such, dental phobia was considered to be different from dental fear and anxiety as it could lead to the avoidance of dental treatment, even when it was necessary [13]. A survey of nearly 2000 adult respondents reported a dental phobia prevalence of 3.7%, a very small number compared to the dental fear prevalence of 24.3% [14]. Dental phobia is not clearly defined in the field of psychology, with some researchers suggesting that it could be an example of a condition called specific phobia, as listed in the Diagnostic and Statistical Manual of Mental Disorders, Fifth Edition (DSM-5) and International Classification of Diseases 11th Revision (ICD-11) [1]. There have also been discussions regarding which subtype of phobia dental phobia should be placed under, with some researchers supporting the blood–injection–injury subtype [15], but with some rejecting this notion and suggesting that it should be an independent subtype by itself [16]. A recent guideline in Germany [17] recommended that a patient should be suspected of having dental phobia and should consult a psychologist or psychiatrist if high levels of dental fear and anxiety are associated with over 2 years of dental avoidance, because these patients may have other psychologic disorders (comorbidity) that are not manageable by dentists alone. Though frequently investigated in dentistry, dental fear, dental anxiety, and dental phobia are not currently explicitly listed in ICD-11 or DSM-5.

Negative health beliefs were reported to directly relate to the increase of dental fear and anxiety [18]. At present, many patients consult online patient education materials for healthcare information, such as YouTube videos [19], with COVID-19 being a prominent example [20]. In particular, dental patients search YouTube videos to seek information regarding various treatment options, such as dental implants [21] and orthodontics [22]. It is equally possible that dental patients who suffer from dental fear and anxiety, being too afraid of seeking help from dentists, might search for YouTube videos to better understand their dental fear, dental anxiety, or dental phobia, and to find ways to manage it. Dental fear, anxiety, and phobia have different etiologies, diagnostic criteria, and manifestations, all of which may determine the relevance/correctness of the information received by the patients. Unfortunately, no prior study has evaluated the content of the YouTube videos dealing with this topic. It is largely unclear who uploaded these videos, what contents are covered, what management methods are introduced, and whether they contain any misleading information. Therefore, the aim of this study was to conduct a qualitative content analysis of YouTube videos on dental fear, dental anxiety, and dental phobia, so that a general overview of the videos concerned could be obtained. The objectives of this study were to reveal the video metrics (e.g., view count), source of the video (whether the channel was by health professions), and aspects of the conditions covered (e.g., prevalence, etiology, symptoms, triggering stimuli, management methods).

## 2. Materials and Methods

Ethical approval was not applicable to the current study.

### 2.1. Information Sources, Search Strategy, and Eligibility Criteria

A search of YouTube videos was conducted on 25 October 2022. The search string in this study was adapted from a qualitative study of YouTube videos related to dental fear and anxiety in children and adolescents conducted by Gao et al. (2013) [23]. Qualitative analysis has often been used in dental research [24]. The terms dental fear, dental anxiety, and dental phobia were searched on the YouTube home page. The first 100 most widely viewed videos that resulted from each search term were recorded and screened. Hence, a total of 300 videos were correspondingly screened for eligibility. Videos with a title or content covering information related to dental fear, dental anxiety, or dental phobia were included. Exclusion criteria included irrelevant (e.g., cartoon, game, and role play video with no information or unrelated to dental fear and anxiety; purely for entertainment; and purely music with/without narrative for meditation or hypnosis purposes) and non-English videos.

### 2.2. Video Selection

The screening of eligible videos was conducted within 14 days, given that a large number of videos are created and uploaded daily and the dynamic nature of social media means it changes rapidly [25]. To achieve objectivity, the titles and content of YouTube videos were reviewed by two independent investigators (NW and AY). Disagreements were resolved by discussion and reaching consensus.

### 2.3. Data Extraction

Two investigators (NW and AY) independently and carefully watched each of the selected videos and conducted a qualitative content analysis of them. One investigator was a PhD student with a psychology background (NW) and one investigator was a dentist experienced in evaluating YouTube videos related to dentistry (AY). The following parameters were extracted and coded for each of the analyzed videos: YouTube video metrics (e.g., number of views, likes, comments, subscriber, duration of video, and uploaded date), characteristics of video (e.g., informants, sources of video, and whether the video contain commercial information), and video content (e.g., information about dental fear, dental anxiety, dental phobia, as well as corresponding treatment or management). Thematic content analysis was applied, and no data analysis software was used [23]. Data were coded as either in vivo codes (words from the informants) or in vitro codes (words and concepts from the authors’ discipline). Codes with similar content were subsequently grouped and merged into analytical categories. Disagreements were resolved through discussion.

### 2.4. Statistical Analysis

Pearson’s correlation tests were conducted to evaluate if there were correlations between the view count, like count, comment count, video duration, and channel subscriber count. Chi-squared tests were conducted to evaluate if the proportion of videos with misinformation was significantly different between video groups, in terms of the video source (lay public, professions versus others), informant identity (health professions versus non-health professions), and view count (high view versus low view, binarized by median split). Statistical tests were conducted using SPSS 26.0 (IBM, Armonk, NY, USA). Results were considered as statistically significant if *p* < 0.05.

## 3. Results

### 3.1. Video Metric

**Figure 1:**
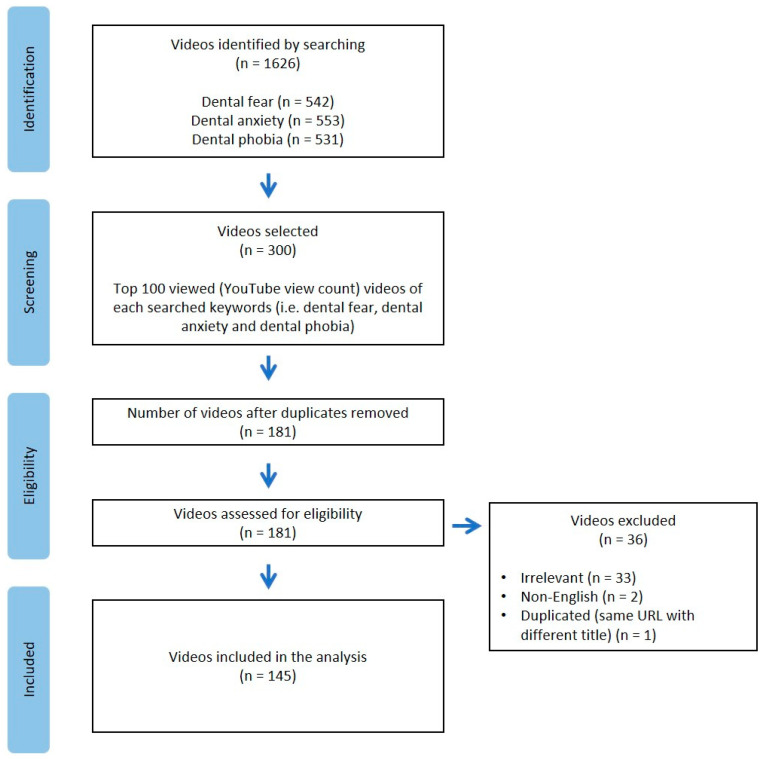
Flowchart of YouTube video screening and selection process.

A total of 1626 videos were identified from the searches. Of the 300 videos screened, 181 videos were recorded and screened for eligibility after removal of duplicates. Videos with irrelevant content (i.e., no information about dental fear, dental anxiety, or dental phobia) or not in English were excluded. A total of 145 videos were included in the final analysis. A flowchart of the video selection and screening process is shown in Figure 1. Agreement between two reviewers was reached in the phase of assessing eligibility, with Kappa coefficient levels of agreement of 94.7%.

**Figure 2:**
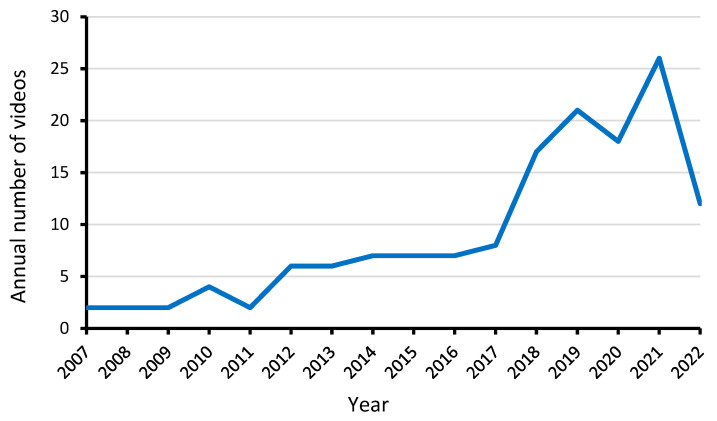
Frequency count of videos uploaded on YouTube from 2007 to 2022.

The frequency count of 145 videos uploaded on YouTube is shown in Figure 2. The oldest video was uploaded in March 2007, while the most recent was uploaded in September 2022. Over 70% (n = 100) of the videos had been uploaded in the past 5 years (2017–2022).

The 145 videos were viewed 139,138 times on average (Table 1), or 28,157 times on average if an outlier with 16,394,106 views was not counted. About 36% (n = 53) of the videos had been viewed over one hundred thousand times, and one of the videos had more than ten million views. The average length of video was 398 s (6–7 min), ranging from 15 s to 40 min 23 s. Each video had approximately 1253 likes, 427,695 subscribers, and 164 comments on average. The comment function was turned off for 15 videos. Like count and comment count were positively correlated with view count, while comment count were also found to be positively correlated with the like count (Table 2).

### 3.2. Sources of Video

**Figure 3:**
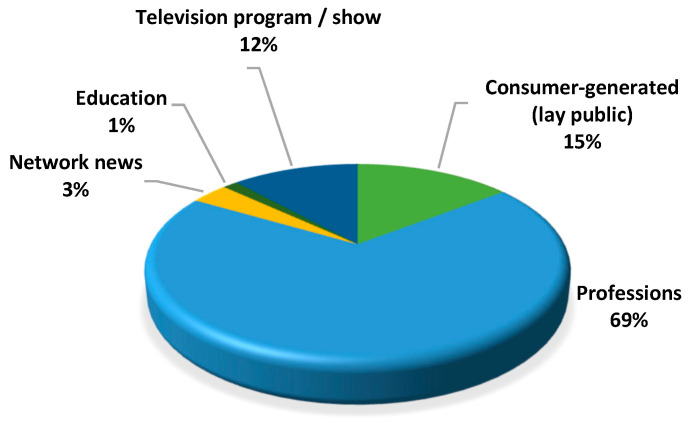
Source of videos on dental fear, dental anxiety, and dental phobia.

Among the included videos, most were created by health professions (*n* = 100; 69%) such as a dentist, dental hygienist, psychologist, etc. The second most common video source was from the lay public (*n* = 21; 15%). The rest were generated from television programs (*n* = 17; 12%), network news channels (*n* = 5; 3%), and the education sector (*n* = 2; 1%) (Figure 3).

**Figure 4:**
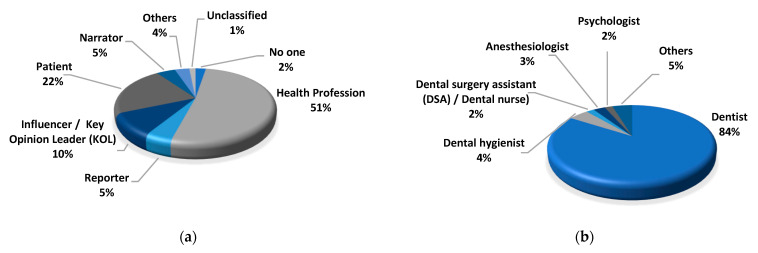
Percentage of informants included in YouTube videos: (**a**) percentage of informants in different categories; (**b**) percentage of health professional informants in different categories.

Figure 4a shows the percentage of informants in different categories. More than half of the informants were health professionals (*n* = 100; 51%), followed by patients (*n* = 42; 22%), and influencer/key opinion leaders (*n* = 19; 10%). News reporters (*n* = 9; 5%) and channel narrator (*n* = 9; 5%) together accounted for 10%. Very few informants (*n* = 7; 4%) were researchers or television hosts. Among those health professions who provided information in the videos, more than three-quarters were dentists (*n* = 90; 84%). Few of the health professions informers were dental hygienist (*n* = 5; 4%), anesthesiologist (*n* = 3; 3%), dental surgery assistant/dental nurse (*n* = 2; 2%), and psychologist (*n* = 2; 2%). The rest (*n* = 5; 5%) included hypnotherapists, physical therapists, psychotherapists, and therapists with an unclear context (Figure 4b).

### 3.3. Video Content Related to Dental Fear, Dental Anxiety, and Dental Phobia

**Figure 5:**
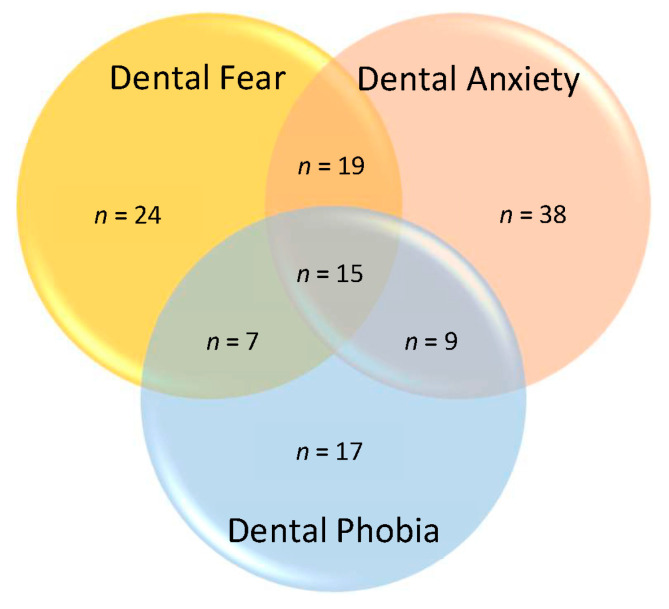
Venn diagram showing how many of the 145 videos mentioned “dental fear”, “dental anxiety”, and “dental phobia”.

Overall, 24 out of 145 videos (16.6%) mentioned dental fear, 38 videos (26.2%) mentioned dental anxiety, and 17 videos (11.7%) mentioned dental phobia. Around one quarter of videos (*n* = 35; 24.1%) mentioned more than one term, and only 15 videos (10.3%) mentioned all three (Figure 5). The content of 16 videos (11%) did not use these terms but used more generic phrases such as “fear of (going to) the dentist”. Among those 50 videos that discussed two or more topics, only three videos described and compared the differences between dental fear, dental anxiety, and dental phobia.

Table 3 shows the general information of the dental fear, dental anxiety, and dental phobia-related content in the 145 videos. Less than half of the videos covered etiology (43.5%). Around one quarter talked about symptoms (25.5%) and effects (25.5%). A few videos mentioned the prevalence (13.1%) and provided general description (7.6%). Only one video discussed diagnostic criteria (0.7%).

Table 4 summarizes the etiology of dental fear, dental anxiety, and dental phobia mentioned in the videos. The causes mentioned in the videos can be categorized into “cognitive factors” and “behavioral factors”. Cognitively, most patients perceived themselves as having no control over the treatment process. Furthermore, patient fears were caused by conditioning. Most patients were behaviorally conditioned by their own unpleasant experiences, by observing and modeling others’ bad experiences, and by hearing others’ experiences.

The symptoms of dental fear, dental anxiety, and dental phobia discussed in the videos are summarized in Table 5. Generally, the symptoms mentioned in the videos could be categorized into “physiological responses”, “behavioral responses”, and “cognitive responses”. Most patients appeared to have a fight-or-flight response, an automatic physiologic reaction to stressful situations, such as increasing heart rate, blood pressure, and sweating. Some patients even had dizziness, stomachache, and vomited when they were going to have or experienced a dental treatment. Furthermore, most patients with dental fear and anxiety were unable to sleep well at night or had nightmares. Some of them had difficulty or hesitation in making dental appointment. Meanwhile, avoidance behavior was shown in patients with dental phobia. Patients with dental phobia would search for alternatives to tackle their dental problems, so as to reduce the need to have a dental visit. They avoided going to a dental clinic until they could not handle oral problems themselves or when they could no longer bear the pain caused by bad oral health. Lastly, patients in the videos claimed that they had irrational thoughts on dental visits. Examples of patients’ narratives are shown in Table 5.

Patients in the videos reported that their fear came from various stimuli. The stimuli mentioned in the videos were mainly classified into (a) dangerous/life-threatening/body injury stimuli; (b) neutral stimuli; and (c) other psychosocial factors. Some examples are summarized in Table 6.

### 3.4. Video Content Related to Management of Dental Fear, Dental Anxiety, and Dental Phobia

**Figure 6:**
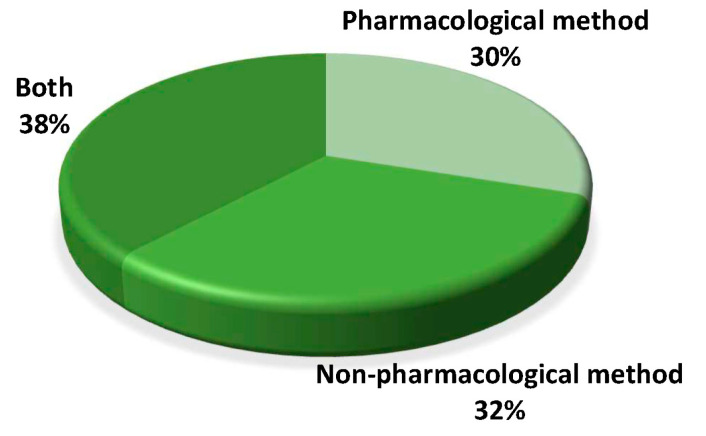
Type of management mentioned in the videos.

Of the 145 videos, only 100 videos (69%) recommended a treatment or suggested interventions to manage dental fear, anxiety, or dental phobia. Among those 100 videos with treatments suggested, 30% mentioned pharmacological methods, while 32% talked about non-pharmacological methods (Figure 6). Meanwhile, 38% discussed both pharmacological and non-pharmacological interventions.

Regarding pharmacological methods, oral medication and intravenous sedation were most commonly mentioned in the videos (18.6%) (Table 7). Some unclarity was found, as supplements with an unclear content were mentioned in two videos (1.4%), and 12 videos (8.3%) mentioned sedation without specifying which type(s). Regarding the non-pharmacological methods, distraction was the most frequently covered specific concept (*n* = 51, 35.2%), which included various methods utilizing audio and visual stimuli (Table 8). Some methods generally used in dental offices were also frequently mentioned, such as signaling and tell-show-do.

Apart from the pharmacological and non-pharmacological interventions reported in the videos, some comment strategies for both patients and dental practitioners in managing dental fear, dental anxiety, and dental phobia were also discussed. Some examples are summarized in Table 9.

### 3.5. Features of Video Presentation Format and Content

The presentation format of the videos is shown in Table 10. All videos were presented with audio. Most videos had a verbal explanation. However, the majority of the videos did not have any text assistance or have a patient demonstrating the management techniques.

**Figure 7:**
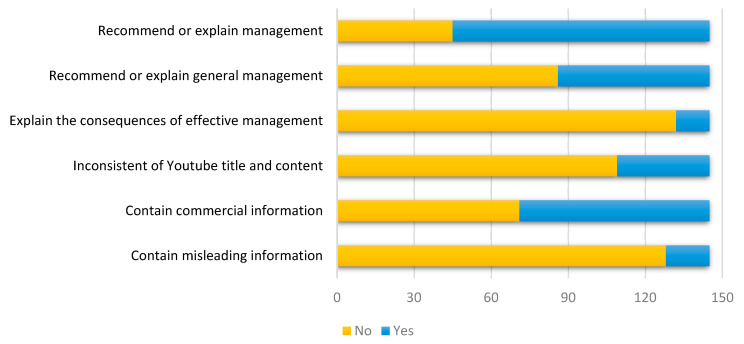
Features of the video content.

Overall features of the videos are shown in Figure 7. Of the 145 included videos, suggestions for managing dental fear, dental anxiety, or dental phobia with pharmacological or non-pharmacological interventions were found in 100 videos (69.0%), while 59 videos (40.7%) provided general strategies for tackling patients’ fear. However, only few videos (*n* = 13; 9.0%) explained and discussed the consequences of effective management of dental fear and anxiety. Moreover, inconsistencies between the video title and content were found in 36 videos (24.8%). Furthermore, more than half of the videos (*n* = 74; 51.0%) contained commercial information (i.e., address, e-mail address, telephone, and fax number of a dental clinic), and 17 videos (11.7%) contained misleading information. The Cohen’s kappa of whether the included videos contained misleading information was 93%.

### 3.6. Video Statistics

There was no significant between-group differences in the prevalence of misleading information in terms of the source of video (*p* = 0.158) and informant in the video (*p* = 0.684) (Table 11). However, the high view group had a larger proportion of videos (19.4%) that contained misleading information compared with the low view group (4.1%), (*p* = 0.004).

## 4. Discussion

This study on YouTube videos about dental fear, dental anxiety, and dental phobia found that the majority had been viewed 1000–100,000 times, implying that they could potentially reach a broad audience on the Internet. Most of them were produced by the professions, with the health professions delivering narratives or patients giving testimonials.

One prominent issue identified was the lack of definition of dental fear, dental anxiety, and dental phobia, or an explanation of their diagnostic criteria. In healthcare, an accurate diagnosis is crucial for proper patient care and management. For example, it is useless to prescribe antibiotics if a patient has a viral infection. Similarly, if a patient complains of oral pain, a dentist should confirm the root cause of the pain before suggesting a treatment. If the pain is due to dental caries, the treatment should be the removal of the decayed part of the offending teeth. In this case, it is useless to advise the patient to try to use mouthrinse more frequently to kill bacteria in the oral cavity. Unfortunately, the analyzed YouTube videos in this study seldom provided a description of what dental fear, dental anxiety, and dental phobia were, or provided diagnostic criteria. There are many psychometric tools devised to assess the dental anxiety level of patients and they are heavily used in academia for benchmarking populations or for diagnostic purposes in research studies, such as the DAS and DFS. However, none of these were introduced in the analyzed videos. In fact, only one video asked the audience four questions from an unclear source, and claimed that the audience might have dental anxiety if their answered yes to at least two questions. Without a proper definition or diagnostic tools on dental fear, anxiety, and phobia, it might be very difficult for the audience to determine if the information provided by the videos was suitable or relevant. It should be noted that not all interventions are suitable for different patients with different anxiety levels and etiology, not to mention that some videos confused or did not differentiate between dental fear, anxiety, and phobia. For instance, most videos proposed sedation (or so-called “sleep dentistry”) as one of the options for patients with dental fear, dental anxiety, or dental phobia. However, sedation cannot help patients to overcome fears and anxiety, as it only allows patients to be put into sleep, so that the dentist can perform dental treatment in a “smoother” manner. Fear and anxiety may still exist without any desensitization at all. Moreover, sometimes it is not practical to put patients to sleep, as some dental procedures, such as one or two simple restorations, can be completed within a relatively short time.

The authors initially intended to evaluate the quality of the videos using existing scales/measurement tools for assessing online patient education materials, such as the DISCERN [29] and JAMA benchmark tools [30]. However, it has been argued that these tools were designed to assess written information and websites but not videos (see Azer, 2020 [31]). For instance, DISCERN requires the assessor to evaluate whether the aims of the assessed material are clearly stated or not. This is not perfectly suitable for assessing YouTube videos, as they are not necessarily produced as educational materials with learning aims and objectives listed at the beginning. In fact, there is a chance that a patient may choose to skip the video if its opening explains its aims in a didactic manner, rendering it boring. A recent systematic review pointed out that studies analyzing YouTube videos for patient education shared common goals of discovering what health-related content the videos contained and the content credibility, but without a standardized set of evaluation method and tools [32]. Perhaps research experts should devise a standard evaluation tool for assessing online videos in the future. Therefore, the authors opted to evaluate the content of the YouTube videos based on items specifically related to dental fear, anxiety, and phobia.

To the authors’ surprise, it was found that the high view videos had a larger ratio of misinformation. This was an alarming finding, as it implied that misinformation could potentially spread across a broad audience. Here is some of the notable misleading information identified from the analyzed videos:

- Misinformation: People with dental fear or anxiety being exposed to videos of dental procedures would be beneficial.
  *Truth: Not everyone would have their dental fear or anxiety alleviated after watching such videos. See [33]. Some might become more anxious.*
- Misinformation: All psychological and non-pharmacological treatments were grouped as “cognitive-behavioral interventions” (CBI).
  *Truth: CBI is a psychological intervention.*
- Misinformation: Dental fear and dental phobia are the same thing.
  *Truth: They differ in severity and phobia leads to avoidance behavior.*
- Misinformation: “Specific dental phobia”.
  *Truth: Such a term has yet to be established in the literature.*
- Misinformation: A video title said there were new drugs that could help dental anxiety.
  *Truth: The drug actually replaced injection (helped with anesthesia) without targeting anxiety.*
- Misinformation: Dental phobia is actually a diagnosis in the Diagnostic and Statistical Manual (DSM).
  *Truth: Some people believed that dental phobia could be an example of specific phobia listed in DSM, but dental phobia itself is not listed in DSM.*
- Misinformation: Laughing gas is not sedation.
  *Truth: Laughing gas (nitrous oxide) is used for inhalation sedation.*

It was unsatisfactory to see such misleading information contained in the videos. Unlike dental treatment topics, the misleading information might not be rectified or clarified by the dentist when a patient went to the dental office for a consultation. Here, patients with dental fear, dental anxiety, or dental phobia might not be as communicative when they go to see a dentist, or they might avoid going to the dentist at all. This implies that it would be much more difficult to correct the misleading information received. Indeed, there have been cases where misleading health information online led to delays in seeking treatment or to receiving harmful/deadly treatment [34]. How the mass media influences the audience has been a recurring research theme for many decades, with multiple theories proposed. For example, the “magic bullet theory” or “hypodermic effects theory” implies that the audience would simply be persuaded and accept the messages received. On the other end, there is the “active audience theory” that implies the audience becomes actively involved in assimilating the messages into their personal and social contexts (for a comprehensive account on the evolution and spectrum of mass media effect theories, please refer to [35]). How dental patients perceive the messages conveyed in these YouTube videos on dental fear, dental anxiety, and dental phobia is largely unknown and should be further examined in future studies. However, it could be argued that patients with heightened dental fear or anxiety may not remain calm and critically analyze the content of these videos, and thus it is important to the include correct information in these videos. It is important for the videos to provide accurate information, as not only dental patients but also dental students may consult them as a learning tool. In fact, it was found that over 95% of dental undergraduates [36] and postgraduates [37] would watch a relevant YouTube video before attempting a clinical procedure.

Despite some notable misleading contents being found in some videos, there were still some potential merits to the videos, as some of them mentioned that their recommendations were based on research findings. Unfortunately, they did not clearly mention their source of information, so that we could not verify whether they had interpreted research findings accurately.

## 5. Conclusions

This report analyzed 145 YouTube videos on dental fear, dental anxiety, and dental phobia. It was found that most were produced by the professions, such as dental clinics and hospitals, with health professions delivering the key messages or with patients giving testimonials. Many etiological factors and symptoms were described. Many pharmacological and non-pharmacological interventions were recommended to the audience, such as sedation and distraction, respectively. However, there was a lack of information on the definition or diagnostic criteria of dental fear, dental anxiety, and dental phobia. Misleading information was found among the videos, and videos with high views had a higher ratio of misleading information. Correct diagnosis leads to proper treatment/management. Without adequate information on how to diagnose, it would be very difficult for the audience to determine if the video content was relevant or useful. Future videos should cover diagnostic criteria, which implies that the dental and psychology fields should work together to properly align the commonly used terms, namely dental fear, dental anxiety, and dental phobia, in DSM-5 and ICD-11.

## Figures and Tables

**Table 1:** Viewing metrics of the 145 videos.

Metric	Mean (SD)	Min; Max	*n* (%)
View count	141,025.68 (1,352,257.81)	740; 16,394,106	
≤1000			5 (3.5%)
1001–10,000			87 (60.0%)
10,001–100,000			45 (31.0%)
100,001–1,000,000			7 (4.8%)
1,000,001–10,000,000			0 (0%)
>10,000,000			1 (0.7%)
Like count	1253.8 (8707.04)	0; 95,000	
≤100			104 (71.7%)
101–1000			32 (22.1%)
1001–10,000			6 (4.1%)
>10,000			3 (2.1%)
Comment count	164.72 (1246.61)	0; 14,052	
≤100			118 (81.4%)
101–1000			9 (6.2%)
1001–10,000			2 (1.4%)
>10,000			1 (0.7%)
Function turned off			15 (10.3%)
Channel subscriber count	427,695.93 (3,174,516.33)	0; 36,600,000	
≤100			30 (20.7%)
101–1000			26 (17.9%)
1001–10,000			33 (22.7%)
10,001–100,000			32 (22.1%)
100,001–1,000,000			20 (13.8%)
1,000,001–10,000,000			3 (2.1%)
>10,000,000			1 (0.7%)
Duration (s)	398 (503.6)	15; 2423	
≤100			35 (24.1%)
101–1000			95 (65.5%)
>1000			15 (10.4%)

**Table 2:** Pearson correlation between video metrics.

Metric	Like Count	Comment Count	Duration (s)	Channel Subscriber Count
View count	0.916 ** (*p* < 0.001)	0.987 ** (*p* < 0.001)	−0.049 (*p* = 0.560)	−0.004 (*p* = 0.965)
Like count		0.910 (*p* < 0.001)	−0.059 (*p* = 0.483)	0.017 (*p* = 0.843)
Comment count			−0.035 (*p* = 0.694)	0.021 (*p* = 0.817)
Duration (s)				0.050 (*p* = 0.554)

** Correlation is significant at the 0.01 level.

**Table 3:** General information on dental fear, dental anxiety, and dental phobia.

	No. of Videos (*n*)	% (of 145 Videos)
Prevalence		
Yes	19	13.1%
No	126	86.9%
Description		
Yes	11	7.6%
No	134	92.4%
Diagnostic criteria		
Yes	1	0.7%
No	144	99.3%
Symptoms		
Yes	37	25.5%
No	108	74.5%
Etiology		
Yes	63	43.5%
No	82	56.5%
Effect		
Yes	37	25.5%
No	108	74.5%

**Table 4:** Summary of etiology/cause of dental fear, dental anxiety, and dental phobia mentioned in the videos.

Categories	Themes	Examples
Cognitive Factors		
	Perceived lack of control	“Most adults also fear a lack of control” “I don’t like the feeling of being out of control” “The dentist made me feel powerless” “I feel helpless in the dental chair”
Behavioral Factors		
	Conditioned by direct trauma	“Unpleasant childhood experience in the dental chair” “Traumatic experience” “When the dentist leans the chair back, some bad experiences popped up” “When I walk to the dental office, the noise of the drill throws me off and it’s scary”
	Conditioned by modeling	“My sister was crying on the dental chair” “They may learn the behavior from observing their parents”
	Conditioned by verbal instruction	“Heard a story about other people’s bad experiences” “My parents told me that my uncle died at the dentist. He was given some anesthetic and he never woke back up” “There were plenty of absolute horror stories tell you how everything went wrong and that doesn’t exactly encourage you to make an appointment”

**Table 5:** Summary of symptoms of dental fear, dental anxiety, and dental phobia mentioned in the videos.

Categories	Themes	Examples ^a^
Physiological Responses		
	Heart rate increase	“My chest feels lie it closes up” “Pounding in my chest” “Racing heartbeat”
	Blood pressure increase	“Their blood pressure will increase”
	Breathing rate increase	“Breath heavily” “Feelings of suffocation” “I need to work out my breathing and calm myself”
	Sweating	“I was sweating” “Sweaty palms”
	Shaky	“I was shaky when doing so”
	Stomachache	“Pit in my stomach” “Pitting of stomach that butterfly kind of feeling”
	Nausea	“Feeling nauseous” “Urge to gag or vomit before dental treatment”
	Dizziness	“Feel dizzy”
Behavioral Responses		
	Avoidance of dental visit	“They always schedule appointment but not show up” “I avoid call to the dentist, it was stressful for me” “Delay going to the dentist” “They don’t often present to a dentist until they have a problem”
	Search for alternative	“Taking over-the counter pain medications to manage the pain”
	Hesitation	“I stand in front of the door of the dental office but not going in” “Debating whether or not I should head back”
	Being sleepless	“I’m not able to sleep” “Sleep badly”
	Nightmares	“I have nightmares from the dentist”
	Extreme behaviors	“I grabbed the dentist hand to stop him” “I wasn’t flossing every day, because I found that even flossing made me kind of look in my mouth and think about my dental state” “Refuse to be reclined in the dental chair”
	Crying	“I sit in the car and cry, because being there made me so afraid”
Cognitive Responses		
	Irrational thought	“I found that even flossing made me think about the dentist, I know it is irrational” “I was feeling vulnerable and thinking I’m going to die”

^a^ Examples from both patients and dental professions.

**Table 6:** Types of stimuli that become feared, as mentioned in the videos.

Categories	Stimuli
Dangerous/life-threatening/body injury stimuli	Needle Sound of the drill Injections Pain Numb Blood
Neutral stimuli	Dental chair Smells of dental office Dental equipment Dentist (white coat syndrome)
Psychosocial factors	Unpleasant childhood experience Stories about other people’s bad experiences Embarrassment Being judged by the dentist (Judgment) Fear of unknown/uncertainty/lack of control Anxiety issue from parents

**Table 7:** Type of pharmacological method mentioned in the videos.

Type of Pharmacological Method	*n*	% (of 145)
Oral medication	27	18.6
Intravenous sedation	27	18.6
Inhalation sedation	24	16.6
Local anesthesia	12	8.3
Just sedation (did not mention which type of sedation)	12	8.3
General anesthesia	11	7.6
Supplements	2	1.4

**Table 8:** Types of non-pharmacological method mentioned in the videos.

Types of Non-pharmacological Method ^a^	*n*	% (of 145)
Distraction	51	35.2
Listen to music (*n* = 24)		
Watch TV or video (*n* = 19)		
Virtual reality (*n* = 5)		
Listen to audio book (*n* = 1)		
Listen to a podcast (*n* = 1)		
Play games with smart glasses (*n* = 1)		
Relaxation	16	11.0
Breathing (*n* = 15)		
Muscle (*n* = 1)		
Hypnosis	6	4.1
Meditation	6	4.1
Aromatherapy/essential oil	5	3.4
Enhanced information	4	2.8
Desensitization	2	1.4
General clinical practice	72	49.7
Signaling (*n* = 21)		
Tell-show-do (*n* = 12)		
Sensory-adapted dental environment (*n* = 11)		
With family member/friend accompany (*n* = 8)		
Positive reinforcement (*n* = 7)		
Body contact (*n* = 6)		
Comfort object (*n* = 5)		
Wearing dark glasses (*n* = 2)		
Others ^b^	7	4.8

^a^ Non-pharmacological interventions listed here were covered by previous systematic reviews [12,26,27,28]. ^b^ Other non-pharmacological interventions included: (each n = 1) acupuncture, cognitive behavioral therapy, cranial electrostimulation device, relaxation system (NuCalm), modeling, deep pressure stimulation (weighted blanket), and emotional freedom technique (EFT) tapping.

**Table 9:** General management techniques suggested in the videos.

Categories	Examples
For patients	Communicate openly (figure out what specifically triggered fear) Schedule appointment at an earlier time Avoid researching information on the internet
For dental practitioners	Communication (figure out what specifically triggered fear) Building a trusting relationship Show compassion/empathy/reassurance Discuss with patients before treatment Acknowledge patient’s anxiety Allow patients to ask questions Make general body language Make eye contact Active listening

**Table 10:** Presentation format of videos.

Presentation Format of Videos	No. of Videos (*n*)	% (of 145 Videos)
Audio		
Yes	145	100%
No	0	0%
Verbal explanation		
Yes	139	95.9%
No	6	4.1%
Text assistance		
Yes	36	24.8%
No	108	74.5%
Have patient demonstration of dental fear anxiety phobia management		
Yes	28	19.3%
No	117	80.7%

**Table 11:** Proportion (%) of videos with misleading information according to different video features.

	Groups of Video		
	With Misleading Information (%)	Without Misleading Information (%)	*p*-Value
Source of video			0.158
Lay public	23.8	76.2	
Professions ^a^	9.0	91.0	
Others ^b^	12.5	87.5	
Informant			0.684
Health professions	12.5	87.5	
Non-health professions	10.2	89.8	
Number of views			0.004
High view	19.4	80.6	
Low view ^c^	4.1	95.9	

^a^ Videos created by hospital, dental clinic, or dental health professions; ^b^ Videos from television programs, television shows, network news, and educational settings; ^c^ Video with <5756 views = low view, by median split.

## Data Availability

The analyzed videos are freely available on YouTube.
